# Atypical Hemolytic Uremic Syndrome Post-Kidney Transplantation: Two Case Reports and Review of the Literature

**DOI:** 10.3389/fmed.2014.00052

**Published:** 2014-12-12

**Authors:** Sami Alasfar, Nada Alachkar

**Affiliations:** ^1^Department of Medicine, Division of Nephrology, The Johns Hopkins University School of Medicine, Baltimore, MD, USA

**Keywords:** aHUS, kidney transplant, eculizumab, genetic mutation, recurrence

## Abstract

Atypical hemolytic uremic syndrome (aHUS) is a rare disorder characterized by over-activation and dysregulation of the alternative complement pathway. Its estimated prevalence is 1–2 per million. The disease is characterized by thrombotic microangiopathy, which causes anemia, thrombocytopenia, and acute renal failure. aHUS has more severe course compared to typical (infection-induced) HUS and is frequently characterized by relapses that leads to end stage renal disease. For a long time, kidney transplantation for these patients was contraindicated because of high rate of recurrence and subsequent renal graft loss. The post-kidney transplantation recurrence rate largely depends on the pathogenetic mechanisms involved. However, over the past several years, advancements in the understanding and therapeutics of aHUS have allowed successful kidney transplantation in these patients. Eculizumab, which is a complement C5 antibody that inhibits complement factor 5a and subsequent formation of the membrane-attack complex, has been used in prevention and treatment of post-transplant aHUS recurrence. In this paper, we present two new cases of aHUS patients who underwent successful kidney transplantation in our center with the use of prophylactic and maintenance eculizumab therapy that have not been published before. The purpose of reporting these two cases is to emphasize the importance of using eculizumab as a prophylactic therapy to prevent aHUS recurrence post-transplant in high-risk patients. We will also review the current understanding of the genetics of aHUS, the pathogenesis of its recurrence after kidney transplantation, and strategies for prevention and treatment of post-transplant aHUS recurrence.

## Introduction

Atypical hemolytic uremic syndrome (aHUS) is a rare disorder characterized by over-activation and dysregulation of the alternative complement pathway. Its estimated prevalence is 1–2 per million (1). The disease is characterized by thrombotic microangiopathy (TMA), which causes anemia, thrombocytopenia, and acute renal failure. aHUS has more severe course compared to typical (infection-induced) HUS and is frequently characterized by relapses that leads to end stage renal disease (ESRD). For a long time, kidney transplantation for these patients was contraindicated because of high rate of recurrence and subsequent renal graft loss. The post-kidney transplantation recurrence rate largely depends on the pathogenetic mechanisms involved. However, over the past several years, advancements in the understanding and therapeutics of aHUS have allowed successful kidney transplantation in these patients. Eculizumab, which is a complement C5 antibody that inhibits complement factor 5a (C5a) and subsequent formation of the membrane-attack complex (MAC), has been used in prevention and treatment of post-transplant aHUS recurrence. In this paper, we present two new cases of aHUS patients who underwent successful kidney transplantation in our center with the use of prophylactic and maintenance eculizumab therapy that have not been published before. The purpose of reporting these two cases is to emphasize the importance of using eculizumab as a prophylactic therapy to prevent aHUS recurrence post-transplant in high-risk patients. We will also review the current understanding of the genetics of aHUS, the pathogenesis of its recurrence after kidney transplantation, and strategies for prevention and treatment of post-transplant aHUS recurrence.

## Cases

### Case 1

In 2011, a 25-year-old Caucasian female developed ESRD due to aHUS. Her initial symptom was shortness of breath and she was found to have severe hypertension, acute kidney injury with serum creatinine of 11.4 mg/dl, anemia with hemoglobin of 5 g/dl, elevated LDH, and thrombocytopenia with platelet count of 20,000/dl. Additional work-up revealed schistocytosis on peripheral smear. She underwent kidney biopsy that revealed TMA involving glomeruli and small vessels. A diagnosis of thrombotic thrombocytopenic purpura (TTP)/HUS was initially made. She received high-dose pulse steroids and four sessions of plasmapheresis. Despite therapy, the disease continued to progress and the patient became hemodialysis dependent. One year later, she was evaluated in our institution for kidney transplantation. Our work-up included ADAMTS13 level, which came back normal and genetic analysis, which confirmed a mutation (c.1933dup A) of complement factor-H (CFH), a variant in exon 13 (SCR11) of the gene CFH. This variant has been identified before and most likely associated with aHUS (personal communication with the Molecular research renal laboratory at the University of Iowa). In mid-2014, she underwent living unrelated kidney transplant. Twenty-four hours prior to transplantation, she received a dose of eculizumab 1200 mg intravenously (IV). Immunosuppression consisted of induction therapy with four doses of intravenous thymoglobulin and 4-day methylprednisolone taper, and maintenance therapy with tacrolimus, mycophenolate mofetil, and prednisone. She had immediate graft function and her serum creatinine was 1.0 mg/dl at the time of discharge. After transplantation, she received weekly eculizumab 900 mg IV for 4 weeks started on day 1 post-transplant, and then 1200 mg IV biweekly starting at week 5 post-transplant; we have utilized the eculizumab protocol recommended by the manufacturing company. Serum complement 3 (C3) level was 37 mg/dl (normal 79–152 mg/dl) at the time of the transplantation and increased to 58 mg/dl 3 months post-transplant. Haptoglobin and LDH levels were normal at the time of transplantation and remained normal afterwards. Hemoglobin was 9.5 g/dl at the time of transplantation and increased to 12.5 g/dl few weeks after. Renal allograft function has remained stable with most recent serum creatinine of 1.1 mg/dl.

### Case 2

In 2002, a 23-year-old Caucasian female developed ESRD due to TMA disorder. Her initial presentation included nausea, vomiting, and jaundice. She was found to have evidence of hemolytic anemia with schistocytosis, thrombocytopenia, and acute renal failure. The initial diagnosis was thought to be TTP. She did not undergo a kidney biopsy. She was started on plasmapheresis but her disease did not respond to the treatment. She also received chemotherapy with vincristine with no response. Her hematologic parameters continued to worsen and eventually splenectomy was required. At the time of diagnosis, she was started on dialysis and remained dialysis depended thereafter. In 2003, she received living unrelated donor kidney transplantation. Her maintenance immunosuppressions were prednisone, mycophenolate mofetil, and sirolimus. In 2005, she developed a diarrheal illness, and her laboratory evaluation showed recurrence of thrombotic microangiopathy including hemolytic anemia, thrombocytopenia, and renal failure. At that time, she was initiated on plasmapheresis; however, her allograft function did not respond to the therapy and she was started again on dialysis. With the recurrence of TMA post-transplant, additional laboratory work-up revealed normal ADAMTS13 level and low C3 level. In 2012, she was evaluated for second kidney transplantation in our institution. Genetic testing was performed and revealed that the patient is heterozygous for two mutations in complement factor related proteins (CFHRs) that may cause aHUS. In fact, these two mutations were not identified previously in aHUS database. The mutations were deletion (*CFH* int20br-*CFHR3*) on allele 1 and duplication in (*CFHR1* int4–*CFHR4* block 7/2 prime) on allele 2. In early 2014, the patient received a successful six-antigen match deceased donor kidney transplant. A dose of eculizumab 1200 mg was administered approximately 24 h prior to transplantation. After transplantation, she received weekly eculizumab 900 mg IV for 4 weeks started on day 1 post-transplant, and then 1200 mg IV biweekly started on week 5 post-transplant. At the time of transplantation, her hemoglobin was 11.2 g/dl and haptoglobin level, LDH level, platelet count, and C3 level were all normal. Six months after transplantation, her hemoglobin increased to 13.5 g/dl. Haptoglobin level, C3 level, LDH, and platelets count remained within normal limits. Allograft function has been excellent so far with most recent serum Creatinine of 0.5 mg/dl.

### Etiology

Atypical HUS can be genetic, acquired, or idiopathic, whereas typical HUS (or diarrhea-associated HUS) is usually triggered by infection with certain strains of *Escherichia coli* that produce powerful Shiga-like exotoxins. There are also other forms of HUS that are secondary to other infections such as *Streptococcus pneumoniae* and HIV or secondary to malignancies, drugs, pregnancy, systemic lupus erythematous (SLE), and anti-phospholipid antibody syndrome (2, 3). More recently, HUS has been described in coagulation dysregulation disorders associated with mutations in certain genes; such as the gene encoding diacylglycerol kinase (*DGKE*) (4).

Atypical HUS is less common than other types of HUS and is characterized by a worse outcome (1, 2, 5). Overall, aHUS accounts for 5% of all HUS cases (2). The incidence of aHUS in the United States is approximately 1–2 per million (1). Atypical HUS may develop in patients of any age, although the majority of cases develop in childhood (1, 2). The disease may have a familial or a sporadic pattern. Most of aHUS cases are linked to genetic abnormalities that involve genes encoding regulatory complement factors. The relationship between low-plasma C3 and HUS was first described by Cameron in 1973 (1, 2, 6). Several mutations in genes encoding regulatory complement proteins have been identified over the last decade. Atypical HUS is the result of abnormal activation and dysregulation of the alternative complement pathway.

### Complement system and its regulation

The alternative complement pathway is persistently activated in plasma. This activation is usually minimal and leads to minor attacks on all structures that the final complement product comes in contact with (1, 2, 5, 7). Under normal conditions, this process is very well regulated by regulatory complement factors. The alternative complement pathway is activated by the spontaneous hydrolysis of complement factor 3 (C3). C3 hydrolysis produces the fragment C3b, which binds to complement factor B (CFB), which in turn is cleaved by complement factor D (CFD) to C3bBb, which is called the C3 convertase. This complex generates more C3b through an amplification loop. As the process continues, more C3b is produced, and eventually [(C3b)2 BbP] is formed. This complex acts as the C5 convertase that cleaves complement factor 5 (C5). Cleavage of C5 generates fragment C5b, which activates the terminal complement pathway by combining with complement factors 6, 7, 8, and 9 to form C5b–9, the MAC. MAC is responsible for endothelial cell damage and leads to micro-thrombosis.

To make this process regulated and prevent over-activation of the alternative complement pathway, different regulatory complement proteins are produced and activated under normal conditions (1, 2). Complement receptor 1 (CR1 or CD35), CFH, and decay-accelerating factor (DAF or CD55) compete with CFB in binding with C3b on the cell surface and can remove Bb from a C3bBb complex. This leads to decreased C3 convertase production. The formation of a C3 convertase is also prevented by complement factor-I (CFI) that cleaves C3b into its inactive form. CFI requires a C3b binding protein co-factor such as CFH, CR1, membrane co-factor protein (MCP or CD46), or thrombomodulin (THBD). CFH related proteins (CFHR1–5) comprise a group of five plasma proteins (CFHR1–5), and each member of this group binds to the central complement component C3b (8). The specific role of each CFHR protein in complement regulation remains unclear; however, they probably play an inhibitory role on C3 convertase and C5 convertase activity (8).

### Genetic basis of aHUS

Atypical HUS is the result of abnormalities in the complement regulatory proteins mentioned above. These abnormalities lead to abnormal activation and dysregulation of the alternative complement pathway. Over the last several years, loss-of-function mutations have been identified in CFH, MCP, CFI, and THBD in some patients presenting with atypical HUS (2, 9). Anti-factor-H and I antibodies have also been recognized in other patients, particularly in children. Gain-of-function mutations in C3 and CFB have also been identified in small percentage of patients with aHUS. Overall, mutations in complement regulatory factors or anti-factor-H and I antibodies have been documented in 60–70% of patients with aHUS (2, 5, 9). The remainder of patients with aHUS probably have genetic abnormalities that yet to be identified. Most of the identified mutations have an incomplete penetrance. It is estimated that the penetrance for most of these mutations is about 50% (1, 2, 10–15). Table 1 summarizes the different characteristics of known mutation that have been associated with aHUS.

**Table 1:** **Characteristics of genetic mutations associated with aHUS and risk of recurrence after kidney transplantation without prevention or treatment**.

Mutation	aHUS incidence	Type of deficiency	C3 level	Risk of recurrence post-transplant
CFH	20–30%	70% Qualitative and 30% quantitative	Low in 30% (quantitative deficiency)	70–80%
MCP	10–15%	70% Qualitative and 30% quantitative	Low in 30% (quantitative deficiency)	Low (unless associated with other mutations)
CFI	5–10%	70% Qualitative and 30% quantitative	Low in 30% (quantitative deficiency)	70–80%
CFB	1–4%	–	Always low	100% (only 4 cases reported)
THBD	3–5%	–	Low in 50%	Unknown but probably very low
C3	2–10%	Majority are quantitative	Low in 80%	40%
Anti-CFH Abs	6%	N/A	Low in 40–60%	40–70% Depending on associated mutations
CFHR 1–5	Unknown	Usually qualitative	Normal unless it is associated with other mutations	Unknown
Combined mutations	10–12%	Variable	Variable	Variable

*aHUS, atypical hemolytic uremic syndrome; C3, complement factor 3; CFH, complement factor-H; MCP, membrane co-factor protein; CFI, complement factor-I; CFB, complement factor B; THBD, thrombomodulin; CFHR, complement factor-H related proteins*.

#### Complement factor-H mutations

These mutations are loss-of-function mutations. CFH mutations are the most frequent genetic abnormality identified in patients with aHUS (2). Up until now, eighty seven mutations in CFH associated with aHUS have been identified (16). They are found in 20–30% of aHUS patients (17–19). Seventy-five percent of the patients are heterozygous and the remainder is homozygous. In 30% of the patients, the mutations cause quantitative deficiency in CFH leading to decrease in CFH and C3 levels. The remainder has functional deficiency of CFH. In the latter case, CFH and C3 levels are usually in the normal range (9, 18, 19).

#### Anti-factor-H antibodies

Auto-antibodies to CFH cause functional deficiency of CFH. They bind to factor-H and inhibit its binding to C3b (2, 20, 21). Anti-factor-H antibodies are responsible for 6% of the aHUS cases. Low-C3 levels are observed in 40–60% of patients with anti-factor-H antibodies. The majority of these patients also have other mutations in CFH, CFI, C3, CD46 (22), or mutations in genes encoding CFHR proteins 1–5 such as deletions or rearrangements (8, 22, 23).

#### Membrane co-factor protein mutations

These are loss-of-function mutations. The abnormal MCP binds weakly to C3b and loses its activity as co-factor (1, 2, 9, 17, 24, 25). Thus far, 28 mutations in MCP have been identified as a cause of aHUS (16). MCP mutations account for 10–15% of aHUS cases. They are more common in children than in adults. In up to 75% of the cases, MCP mutations cause low-MCP expression on peripheral leukocytes, which can be used as a diagnostic tool. However, in the remainder of cases, MCP mutations do not cause low-MCP expression (25). Around 30% of patients with MCP mutations have low-C3 level (2, 5, 7, 9, 17, 25).

#### Complement factor-I mutations

These are loss-of-function mutations. CFI mutations account for 5–10% of aHUS cases (21, 26–28). To date, there are 11 mutations in CFI that have been associated with aHUS (16). Similar to CFH mutations, CFI mutations may lead to either a quantitative or functional deficiency in CFI. In 30% of the patients, the mutations cause quantitative deficiency in CFI leading to decrease in both CFI and C3 levels (2, 7, 9, 21, 26–28).

#### Thrombomodulin mutations

These are also loss-of-function mutations. Cells with mutant THBD are less efficient at inactivating C3b. THBD mutations account for 3–5% of patients with aHUS. C3 levels are low in 50% of patients THBD mutations (2, 7, 9, 12, 29).

#### Complement factor B mutations

Complement factor B mutations are usually gain-of-function mutations and result in increased stability of C3 convertase and its resistance to degradation. CFB mutations account for 1–4% of patients with aHUS (7, 30). Patients with CFB mutations always have low-serum C3 level (2, 7, 9, 30–32).

#### Complement component 3 mutations

The mutant C3 binds weakly to MCP and/or CFH, which leads to indirect gain-of-function for C3 activity (7, 33). Therefore, binding of CFB to C3b increases and more C3 convertase is formed. C3 mutations account for 2–10% of patients with aHUS. In 80% of cases, serum C3 level is low (2, 7, 9, 32, 33).

#### Combined mutations

Up to 10–12% of patients with aHUS have two or more mutations in different complement factors (32). This suggests that aHUS may result from the additive effects of several genetic factors (2).

#### Complement factor-H related proteins genetic polymorphism

Genetic polymorphisms in CFH related proteins may create a slight increase in predisposition to aHUS and could influence the severity of disease (7, 8, 26, 34–36). The area of abnormalities in genes encoding CFHR proteins still needs further investigation. Mutations, genetic deletions, duplications, or rearrangements in the individual CFHR genes are associated with a number of diseases including aHUS, C3 glomerulopathy, IgA nephropathy, age related macular degeneration (AMD), and SLE (36). Rearrangement that leads to the formation of hybrid *CFH/CFHR1* gene has been associated with aHUS (7, 37). A hybrid factor-H/CFHR3 gene generated by a microhomology-mediated deletion was reported in a familial aHUS case (38). Deletion of CFHR1/CFHR3 is also strongly associated with the development of factor-H auto-antibodies (36, 39–41). *CFHR1* deficiency alone could modulate the severity of aHUS associated with another mutation (26). Genetic variants of CFHR5 were also reported in aHUS patients (33, 42).

## Risk of Recurrence

Atypical HUS, unlike infection-induced HUS is associated with very high risk of recurrence after kidney transplantation (5, 43). Concern over the recurrence of HUS after kidney transplantation has been recognized for a long time (44, 45). In 2003, Loirat and Niaudet (43) studied the risk of recurrence of HUS after kidney transplantation in children and found that the risk in patients whose original HUS was not associated with diarrhea is higher compared to the infection-induced HUS. Sixty-three children, who had the non-diarrheal HUS, received 77 kidney transplants. Thirteen patients (21%) had recurrence of HUS after one or more grafts, and there were a total of 18 recurrences in 77 grafts (23%). In later observational studies, the recurrence rate of HUS in adult patients who received kidney transplantation was as high as 50–60% (7, 43, 46–48).

Over the past few years, several studies have assessed the risk of post-transplant aHUS recurrence and prognosis associated with specific genetic abnormalities (7, 17, 24, 49, 50). The recurrence rate after transplantation in aHUS patients essentially depends on whether the mutant complement factor is membrane-bound or circulating. Membrane-bound factors expression in the renal allograft is determined by the donor genome. Thus, patients with mutations in genes encoding one of these factors are not expected to develop post-transplant aHUS recurrence. On the other hand, the risk of post-transplant aHUS recurrence in patients with mutations encoding circulating factors is expected to be high (5, 7, 24, 49, 51).

In two French case series (24, 51), the rate of post-transplant aHUS recurrence in patients with *CFH* mutations was around 75–80% in 5 children and 16 adults who received 6 and 17 renal transplants, respectively. Around the same recurrence rate was noted in a 2006 meta-analysis of 36 renal transplantations in 27 patients with aHUS associated with *CFH* mutations (49). Recurrence of aHUS with this type of mutation was associated with poor prognosis, leading to graft loss in 93% of patients (49).

In patients with anti-CFH antibodies, the risk of post-transplant aHUS recurrence is not well known. Studies so far only described a total of 12 kidney transplantations in eight patients (1, 7, 22, 35, 52–54). The assessment of the risk is further complicated by the finding that as many as 40% of patients with anti-CFH antibodies also carried a mutation in genes encoding complement regulatory factors, including *CFH*, *CFI*, *MCP*, and *C3* (22). Initially, it was thought that reduction in levels of anti-CFH antibodies with plasmapheresis and rituximab permitted successful kidney transplantation in these patients, suggesting that high anti-CFH antibody levels positively correlate with risk of post-transplant aHUS recurrence (52, 53). However, later publications have reported that recurrence-free transplantation is achievable in patients with anti-CFH antibodies despite the absence of any specific treatment (22, 53, 54).

In aHUS patients with *CFI* mutations, the post-transplant recurrence rate is high and associated with poor prognosis (1, 7, 18, 20, 21, 24, 55–58). Available publications reported 10 patients, who received 15 kidney transplants. 12 of 15 (80%) transplants consecutively failed because of aHUS recurrence. In a paper from 2009 (26), it was found that more than half of the *CFI*-mutation patients had an additional genetic susceptibility for aHUS and that those with an isolated *CFI* mutation had improved kidney survival over those with additional mutations. Thus, the post-transplant risk of aHUS recurrence for patients with a *CFI* mutation should be reassessed in the light of this finding.

Atypical HUS patients with MCP mutations are expected to have a low risk of post-transplant aHUS recurrence because MCP is a membrane-bound protein (7, 59). Therefore, the prognosis of patients with *MCP* mutations after kidney transplantation is much better than that of patients with mutations in *CFH* and *CFI* (7, 18, 24, 25, 59, 60). However, a recent large study of aHUS patients revealed the unexpected recurrence of disease after transplantation in some carriers of MCP mutations. This is probably explained by the fact that a significant proportion (22.6%) of patients with mutations in MCP also carried mutations in genes encoding circulating complement factors (61). Notably, post-transplant recurrence occurred in 7.6 and 30% of MCP single-mutated and MCP-combined mutated patients, respectively (61). In our previously published series (62), the only patient who had MCP mutation did not develop disease recurrence after kidney transplantation.

There is very limited data on the post-transplant recurrence of aHUS in patients with *THBD* mutations. THBD exists both in membrane bound and soluble forms, hence, it is not possible to reliably predict the risk of post-transplant recurrence in carriers of these mutations. In the available study (12) that describes seven patients with *THBD* mutations, one individual experienced post-transplant recurrence of aHUS. In another case report (29), a 19-year-old man with aHUS secondary to a THBD mutation relapsed twice after two kidney transplantations performed 12 years apart, which suggests that THBD mutations may favor-relapse of aHUS after kidney transplantation. In our previously published series (62), there was one patient with THBD mutation who developed early recurrence after kidney transplantation.

Atypical HUS patients with C3 mutations have reduced risk of recurrence after transplantation although it remains high after all (7). In a study that reported 12 kidney transplant recipients with C3 mutations, 5 of them (42%) developed recurrence (1, 11). Most circulating C3 is synthesized by hepatocytes; however, smaller though significant amounts are produced in other tissues including the kidney. Therefore, it is possible that production of non-mutant C3 by the graft might account for the reduced recurrence rate (63, 64).

In aHUS patients with CFB mutations, there are four cases reported and in all four transplants failed because of recurrence (7, 10, 65).

## Post-Transplant aHUS Recurrence Triggers

The natural history of aHUS in kidney transplant patients varies from that of non-kidney transplant patients (1, 5, 7). The recurrence of aHUS in transplants patients usually occurs within the first year after transplantation (5, 49, 51). This is different from non-transplant patients in whom the onset of aHUS is frequently delayed, with up to 50% of cases presenting in adulthood (5, 66). This difference is probably driven by the environment that kidney transplantation creates, in which multiple potential triggers for aHUS recurrence exist. A meta-analysis in 1998 (67) attempted to identify clinical risk factors associated with high risk of aHUS recurrence in post-transplant patients. The study concluded that old age at presentation, short duration between onset of disease and ESRD or transplantation, use of living donors, and use of calcineurin inhibitor (CNI) are all associated with high risk of recurrence. It has been suggested that recurrence after transplantation may be less common among patients who have undergone a nephrectomy prior to transplantation. However, there is only once study that reported this observation. In this study, pooled data from several series was analyzed, and recurrence was reported among 5 of 14 patients who had undergone nephrectomy compared with 27 of 35 patients who had not undergone nephrectomy (47).

There are different mechanisms in the kidney transplantation process that may trigger recurrence of aHUS including brain-death related injury, ischemia–reperfusion injury, infections, the use of immunosuppressive drugs, and rejection (5).

There is growing evidence about the role of complement activation in brain-death-induced organ injuries (5, 68, 69), which could be a trigger for aHUS recurrence. In brain death, there is an increased circulating C5a level that induces a release of pro-inflammatory cytokines by the kidney and eventually leads to increased formation of C5b–9 complex.

Ischemia-reperfusion leads to endothelial damage in kidney allograft. Studies proposed that this damage causes reduced CFH ability to bind to the endothelium (70), ongoing alternative pathway activation, and increased formation of C5b–9 (5). Given this possible role of complement activation in the pathology of delayed graft function, there are several clinical trials that are underway to assess the efficacy of eculizumab in prevention of delayed graft function in deceased donor kidney transplantation (http://www.Clinicaltrials.gov NCT01919346).

Many infections have been involved in triggering post-transplant aHUS recurrence (5). These include CMV (71), influenza virus (72–74), parvovirus B19 (75), BK virus (76), upper respiratory tract infection (19, 77), and infectious gastroenteritis (19, 78).

Also, immunosuppressive drugs could trigger post-transplant aHUS recurrence (2, 5, 7). Both calcineurin- and mTOR inhibitors have been associated with endothelial toxicity (79, 80) and post-transplant TMA. Prolonged CNI-related ischemia is believed to induce endothelial damage and initiate the pathogenic processes involved in the development of TMA (7, 81). Despite the general idea that CNI play a prominent role in stimulating post-transplant aHUS, the only available study that assessed the risk of aHUS recurrence with different drugs showed that mTOR inhibitor-based regimens only were found to be an independent risk factor for post-transplant aHUS recurrence (51). In contrast, this study failed to identify CNI-based regimens as risk factors for post-transplant aHUS recurrence. In addition, other studies found that mutations in CFH and CFI genes were identified in 30% of transplant recipients with CNI-induced *de novo* post-transplant aHUS (5, 7, 82). This suggests that factors, resulting in complement over-activation may be required for CNI-induced endothelial toxicity to clinically manifest. Interestingly, in the context of post-transplant CNI toxicity, systemic aHUS, unlike localized renal TMA, usually fails to respond to reduction or temporary discontinuation of CNI therapy (83) and respond dramatically to complement blockade (84). In an attempt to reduce the burden of insults to the graft endothelium, the CTLA-4-Ig fusion protein belatacept, which does not cause any endothelial toxicity, might be a promising alternative to calcineurin and mTOR inhibitors for maintenance immunosuppressive regimens but this drugs needs to be further investigated (7).

Rejections could also trigger post-transplant aHUS recurrence (7). The renal allograft endothelium is the primary target of anti-HLA antibodies. Although antibodies activate the complement cascade primarily through the classical pathway, one can reasonably hypothesize that uncontrolled activation of the alternative pathway in aHUS patients could further amplify the endothelial damages induced by anti-HLA antibodies.

## Clinical Presentation

Patients with post-transplant aHUS usually present with a microangiopathic hemolytic anemia, thrombocytopenia, and acute kidney injury. Typical laboratory abnormalities include an increased serum creatinine; evidence of hemolysis (such as increased reticulocyte percentage, reticulocytosis, schistocytes on peripheral smear, and increased serum lactate dehydrogenase); and a low-platelet count. The urinalysis typically shows hematuria and only a small amount of proteinuria. Patients with post-transplant aHUS recurrence may develop fever but the presence of chills and high-spiking fever should suggest that the presence of infection that could be the trigger of aHUS recurrence. Recurrence could also manifest with neurological symptoms such as confusion and headache. Focal neurological abnormalities (e.g., transient aphasia, transient ischemic attack, and stroke) are less frequent, but grand mal seizures and coma can occur. Imaging studies may show a pattern consistent with reversible posterior leukoencephalopathy syndrome (PRES) (85). Gastrointestinal symptoms of aHUS include abdominal pain, nausea, vomiting, and diarrhea. Cardiac involvement includes diffuse platelet thrombi and associated hemorrhage and patches of necrosis in cardiac tissues (e.g., coronary arteries, myocardium, and conducting system), which may lead to complications such as arrhythmia, sudden cardiac death, and myocardial infarction (86).

Some patients may present with only an increased serum creatinine and abnormal urinalysis due to the renal lesion associated with TMA, and without thrombocytopenia, hemolytic anemia, or other manifestations (45, 87). In 2014, Stevenson et al. (88) described a case of unsensitized aHUS patient who received deceased donor kidney transplant. The patient developed severe antibody mediated rejection (AMR) that was unresponsive to aggressive therapy with evidence of new DSA formation. The rejection led to graft failure. This case suggested that dysregulation of the alternative complement pathway within the transplanted kidney may have contributed to the severe AMR. Licht et al. (89) reported an AMR case, presenting with a systemic thrombotic microangiopathic process, in a kidney transplant recipient harboring a homozygous deletion of CFHR3-1. In this patient, eculizumab rescue therapy rapidly resulted in a complete recovery from the AMR-induced TMA, further supporting the benefit of complement blockade in severe AMR. There is an ongoing trial that is currently underway to study the efficacy and safety of Eculizumab for treatment of AMR following kidney transplantation (http://www.ClinicalTrials.gov NCT01895127).

The differential diagnosis of post-transplant aHUS recurrence after kidney transplantation is similar to that of non-kidney transplant patients. This includes TTP and secondary forms of HUS due to other causes. Shiga toxin-producing *E. coli* (STEC) induced HUS is differentiated from aHUS based on demonstrating a recent exposure to STEC. Thus, screening for STEC should be performed in these cases. *S. pneumoniae* induced HUS occurs in a patient with evidence of a pneumococcal infection (e.g., pneumonia, sepsis, or meningitis), which is confirmed by a positive culture of blood and/or other pertinent tissues. HIV infection should also be excluded. Other rare non-infectious secondary causes of HUS include drug toxicity (examples: quinine, anti-platelet therapy, and chemotherapy), and autoimmune diseases like SLE or anti-phospholipid syndrome. HELLP syndrome and post-partum HUS should be kept on the differential diagnosis if the history is suggestive; although recent data showed that complement mutations were described in 36% of women with HELLP syndrome and 86% of cases of post-partum HUS. Thus, we may conclude that pregnancy could act as a trigger for aHUS rather than a direct cause of it.

## Prevention

For many decades, kidney transplantation was contraindicated for patients with ESRD secondary to aHUS due to the high-recurrence rate. However, over the last decade, perceptions on kidney transplantation in aHUS have changed. Transplantation may now be considered on a case-by-case basis, with individualized strategies based on donor characteristics and recipient genetics findings (5, 7, 90). Certain strategies that primarily target preventing endothelial damage, that could potentially trigger aHUS in renal allograft, can be used to make the kidney transplantation successful. As mentioned earlier, the kidney transplantation process leads to transient or continuous endothelial damage in different mechanisms. This damage may serve as the trigger to alternative complement pathway activation and subsequently recurrence in aHUS. These insults are discussed earlier, and include brain death, ischemia/reperfusion injury, infections, immunosuppressive drugs, and rejection. In theory, strategies that minimize or halt these insults may lead to better renal allograft outcome.

As mentioned earlier, brain death and ischemia-reperfusion play a role in complement activation. Thus, aHUS patients, who are prone to uncontrolled complement activation, may be more susceptible to delayed graft function and ischemia-induced irreversible graft damage (5, 51). Therefore, it seems reasonable to exclude expanded criteria donors with the greatest risk of delayed graft function, such as non-heart-beating donors or those with prolonged cold ischemia times, from donation to aHUS patients. Also, to reduce the burden of post-transplant endothelial damaging factors, renal transplantation across positive crossmatches and/or with preformed donor specific antibody should be evaluated carefully, especially in patients who are highly sensitized and incompatible donation is the only option available for transplantation. In our previously published series, few patients with aHUS received ABO and HLA incompatible kidney transplantation (62).

Systemic infections have been found to trigger aHUS episodes. Thus, infections in kidney transplant recipients with aHUS must be treated aggressively to prevent aHUS recurrence. Particular attention must be paid to preventing CMV infection with adequate prophylaxis in kidney transplant recipients with aHUS (5, 71, 91).

In terms of immunosuppressive drugs, although CNI have been associated with endothelial toxicity (79, 80) and post-transplant TMA, a cohort study failed to identify CNI-based regimens as risk factors for post-transplant aHUS recurrence (51). However, CNI overexposure may trigger aHUS recurrence, emphasizing the need of careful CNI blood levels monitoring. On the other hand, mTOR inhibitors have been independently associated with a greater aHUS recurrence rate, and should not be used in aHUS patients (5, 51). Belatacept therapy that has no risk on endothelial injury should be studied further; it may be considered an alternative maintenance immunosuppressive therapy in these patients (5, 7).

### Type of transplant

The type of transplant may play a role in prevention of post-transplant aHUS recurrence. There are two options for kidney transplantation in patients with aHUS. This includes combined liver–kidney transplantation (CLKT) and isolated kidney transplantation.

#### Combined liver–kidney transplantation

The motivation to treat individuals with ESRD due to aHUS by CLKT is that liver transplant represents the “gene therapy” for those individuals whose aHUS is due to a defect in a circulating product synthesized by the liver. However, the early experiences with CLKT were disappointing. The first five cases did not include preoperative treatment with plasma therapy and had a fatal outcome. In three of these cases, there was evidence of complement-mediated coagulative hepatic necrosis (5, 92–94). As mentioned earlier, it is suggested that with ischemia/reperfusion, significant modification in the endothelial happens leads to complement activation (95, 96). Thus, it was suggested that CLKT should not be done without a preoperative regimen for complement regulation such as plasma therapy, or anti-C5 treatment with eculizumab. Unfortunately, there is no registry that gathers and follows the outcome of aHUS cases that were treated with liver or combined liver and kidney transplantation. A summary of more recent cases that were treated with liver or CLKT was published in 2013 (97). These cases were also treated with preoperative eculizumab or plasma therapy. The overall success rate was 16 out of 20 (80%) and the mortality rate was 3 out of 20 (15%). Out of 20 transplants, 16 were carrier of CFH mutations (14 successful), 1 for CFB (successful), 1 for C3 (unsuccessful), and 2 for CFH/CFHR1 hybrid mutation (1 successful). One successful transplant was an isolated liver in a patient with remaining kidney function before the availability of eculizumab. Plasma therapy alone was used in 18 (15 cases successful) and eculizumab with plasma therapy in 2 (1 successful).

Nevertheless, the risks inherent to the surgery and the safer newly available alternative preventative and therapeutic options have made CLKT less favorable for treatment of aHUS. Thus, the option of CLKT should be pursued on a case-by-case basis, depending on individual’s physical status, local expertise of the transplant surgeons, ability to afford a long-term eculizumab therapy, and patient wishes regarding quality-of-life (5, 90).

#### Isolated kidney transplantation

Over the last several years, advancements in the management of aHUS have allowed successful isolated kidney transplantations in patients with ESRD secondary to aHUS. There is no longer rationale for barring kidney transplantation in aHUS patients from deceased donors and from living non-related donors, provided that anti-C5 treatment with eculizumab is available for preventing or treating aHUS recurrence. Prior to transplantation, many centers screen patients for genetic abnormalities in complement regulatory proteins before inclusion on kidney transplant waiting lists. It is not acceptable to do the distinction between infection-induced HUS and aHUS based on prodromic diarrhea alone, because approximately 20–30% of cases of aHUS are preceded by diarrhea (7, 24). In the case of living donors, most centers screen donors and exclude them if they are found to carry mutations in the above-mentioned genes. Historically, living-related kidney transplantation was contraindicated in aHUS patients in most centers (1) because of the risk of the donor carrying the same mutation that the recipient has. However, this recommendation had changed with the recent approaches that have been used for prevention and treatment of aHUS post-transplantation recurrence.

### Plasma therapy/plasmapheresis

There is limited published data on the efficacy of prophylactic plasma therapy to prevent post-transplant aHUS recurrence. It was first demonstrated to be efficient in preventing post-transplant aHUS recurrence in patients in whom genetic abnormalities predicted a high risk of recurrence following kidney transplantation (5, 51, 53, 61, 91). However, in other studies, prophylactic plasma therapy failed to prevent post-transplant aHUS recurrence in some cases (51, 98). In a small sample study (51), nine patients received preemptive plasma exchange therapy in an attempt to prevent post-transplant recurrence of aHUS. In multivariate analysis, prophylactic plasma therapy decreased graft loss [RR = 0.11 (0.01–0.84) *p* = 0.035]. There was also a non-significant decrease in disease recurrence [RR = 0.34 (0.10–1.13) *p* = 0.078] in patients who received preemptive plasma exchange therapy. In addition to this, some small studies demonstrated that there was an unpredictable risk of recurrence when plasmapheresis sessions were progressively spaced out (5, 59, 91). Lastly, the issues of prolonged adequate vascular access (5), plasma allergy (59), and quality-of-life (99) should also be taken into account when treating with long-term plasma therapy. In antibody-induced aHUS (e.g. anti-CFH antibodies), preventative plasmapheresis is the therapy of choice. In recent papers, attempts to reduce titers of anti-CFH antibody with preemptive plasmapheresis and/or rituximab have enabled successful kidney transplantation in two patients with aHUS-related ESRD (5, 100, 101).

### Eculizumab

Eculizumab is a recombinant humanized monoclonal anti-C5 antibody, licensed initially for the treatment of paroxysmal nocturnal hemoglobinuria (2, 102), was recently approved for the treatment of aHUS (2). Eculizumab binds specifically to the complement protein C5, halting the complement cascade and inhibiting production of cell-killing protein complexes (103).

In 2011, Weitz et al. (77) reported the first successful kidney transplantation in a child with CFH deficient aHUS after the prophylactic administration of eculizumab. In 2012, Zuber et al. (98) in France reported nine patients who received prophylactic eculizumab therapy to prevent post-transplant aHUS recurrence, eight of whom experienced a successful recurrence-free post-transplant course, while one lost his graft from an early graft artery thrombosis. They all harbored a complement genetic abnormality associated with a risk of aHUS recurrence >80%. Recently, our institution (62) reported four patients with aHUS who received prophylactic eculizumab at the time of transplantation. Three of them received eculizumab for 6 months post-transplant and one of them received it as life-long therapy. The four patients did not develop aHUS recurrence. Earlier in this paper, we included two new cases of aHUS caused by CFH and CFHRs mutations that underwent successful kidney transplantation with prophylactic life-long eculizumab.

Overall, these data suggest that long-term eculizumab treatment is highly effective for the prevention of post-transplant aHUS recurrence. More studies are needed to determine the optimal dosing and duration of prophylactic therapy (90).

There are no established guidelines for eculizumab dosing in prevention of aHUS. In our institution, the first dose is administered within 24 h before the operation. Prophylactic strategy should be initiated before the surgery in order to hamper ischemia-reperfusion injury related complement activation. We continue with weekly infusion starting on day 1 post-transplant for 4 weeks, then biweekly infusion. The decision when to stop eculizumab therapy is based on the patient’s mutation type. We currently recommend life-long therapy for patients with mutations that are associated with high-recurrence rate such as CFH, CFI, and CFB mutations.

## Treatment

### Plasmapheresis

Earlier studies did not show good outcome with plasmapheresis alone in the treatment of post-transplant aHUS recurrence. In Noris et al. paper (1), plasma therapy induced remission in 55–80% of episodes in patients with CFH, C3, or THBD mutations or auto-antibodies, whereas patients with CFI (factor-I) mutations were poor responders. In Le Quintrec’s (51) paper, curative plasma therapy, which consisted either of fresh frozen plasma infusions (*n* = 3) or plasma exchanges (*n* = 30), was performed in 33 of the 44 patients (75%) with aHUS recurrence. Overall, graft outcome was poor in patients who received curative plasma therapy for recurrence in univariate analysis [RR = 2.51 (1.34–4.68) *p* = 0.004]. After adjustment for recurrence, multivariate analysis showed that curative plasma therapy did not improve graft survival [RR = 1.17 (0.49–2.83) *p* = 0.7].

### Eculizumab

There are multiple case reports and series that showed success with eculizumab in the treatment of plasmapheresis resistant (104–106) and dependent aHUS forms (99, 104, 106–108). There are also some cases where eculizumab was also used as a first line therapy without any prior plasma therapy (106, 109).

In 2011, Al-Akash et al. (72) reported one case in which eculizumab with plasmapheresis induced long-term remission in recurrent post-transplant aHUS associated with C3 gene mutation. In 2012, we reported a case of two post-transplant aHUS recurrences in a patient with unclassified aHUS. The first recurrence responded to plasmapheresis and eculizumab. The second recurrence was treated successfully with eculizumab alone (104). Zuber et al. (98) also reported thirteen kidney transplant recipients were given anti-C5 for post-transplant aHUS recurrence. A complete reversal of aHUS activity was achieved in all of them. In this case series, it was noted that the delay of anti-C5 initiation after the onset of the aHUS episode inversely correlated with the degree of renal function improvement. Three patients in whom anti-C5 was subsequently stopped experienced a relapse. Recently, we presented our center’s data (62), in which three patients with post-transplant aHUS recurrence after kidney transplantation were treated with eculizumab and plasmapheresis, two of them responded to the therapy. The patient who did not response had CFH mutation.

Legendre et al. (107) have recently published the results of two trials that studied the efficacy of eculizumab in adults and adolescents with aHUS resistant to plasma therapy (trial 1; 17 patients) or on chronic plasma therapy (trial 2; 20 patients). These two trials together included 15 kidney transplant recipients. In both trials, the primary end point (i.e., normalization of platelet count and TMA-free status, respectively) was achieved by more than 80% of the patients. Interestingly, similar to Zuber’s paper, it was noted that there was an inverse correlation between the onset of the aHUS recurrence and eculizumab initiation with the recovery of renal function in both transplanted and non-transplanted patients. In these 2 studies, 5 out of 18 patients who missed eculizumab doses developed post-transplant aHUS recurrence. In two case reports, patients received only a single dose for the treatment of recurrent post-transplant aHUS (104, 105). Although both patients had sustained response with a prolonged aHUS-free period, delayed recurrence occurred in the first patient after 11.5 months (105) and in the other after 21 months (104). The reintroduction of eculizumab led to remission of aHUS in the former patient but failed to prevent graft loss in the later. In another case report, a delay of 6 days in the ninth infusion of eculizumab led to a mild relapse of post- transplant aHUS, but the disease responded rapidly to the re-initiation of treatment (110).

The studies above illustrate the importance of prompt treatment with eculizumab therapy once a recurrence is diagnosed. They also show that maintenance treatment is more effective than single dose therapy in preventing recurrence. There is also a suggestion that more recurrence occurs when administration of eculizumab is spaced out by more than every 2 weeks. Altogether, the available data also illustrate the critical need for prospective well-controlled studies to address the issue of treatment dosing and duration.

### Future therapies

Concentrated CFH, purified from human plasma, may serve as a future therapeutic option for aHUS patients with CFH mutations, especially in patients with a quantitative deficiency in functional CFH (5, 7). However, there are several questions that need to be addressed regarding its therapeutic efficacy, bioavailability, potential dosing, and complications relating to competition between the plasma-derived CFH and the mutated protein. Also, recombinant THBD can be utilized in the treatment of aHUS (12). The use of recombinant human THBD, which also has anticoagulant and anti-inflammatory properties, was approved in Japan in 2008 for the treatment of disseminated intravascular coagulation. Of note, a case of refractory TMA after hematopoietic stem cell transplantation was published in 2009; the TMA was successfully treated with recombinant THBD (111). Other complement-modulating agents under development might represent additional therapeutic avenues (112) and need more investigation.

## Summary

Atypical HUS is characterized by hemolytic anemia, thrombocytopenia, and renal impairment that frequently progresses to ESRD. In more than 50% of patients, mutations in complement regulatory proteins or antibodies to these proteins are identified. Among patients who undergo kidney transplantation, aHUS commonly recurs, especially in patients who carry mutations. Recurrent aHUS after kidney transplantation is associated with high rate of allograft failure. The type of the mutation dictates the overall prognosis and the risk of post-transplant recurrence, which ranges from very minimal up to 80%. Although combined liver and kidney transplantation provides the “gene therapy” for aHUS, it remains a high-risk operation and with the successful use of eculizumab in prevention and treatment of post-transplant aHUS recurrence, this option became less favorable, although it should not be overlooked. Isolated kidney transplantation remains the best option for ESRD due to aHUS. Nonetheless, in the milieu of kidney transplantation, there are several triggers for aHUS recurrence and this includes immunosuppressive drugs, brain-death related injury, ischemia-reperfusion injury, and infections. Strategies that minimize or block these potential triggers could prevent aHUS recurrence. The available published data strongly suggest that the use of prophylactic treatment with eculizumab rather than plasmapheresis alone. With the limited available data, eculizumab should be continued indefinitely. Among all transplantation recipients who develop recurrence in aHUS after transplantation, treatment with eculizumab rather than plasmapheresis alone should be promptly initiated. However, therapy with eculizumab carries its own risks and is associated with meningococcal infections that can be life threatening. Thus, meningococcal vaccination is required in all patients treated with eculizumab. There are several questions regarding the safety, dosing, and duration of prophylaxis and/or treatment with this drug. The best approach to answer these questions can be achieved by a prospective controlled trial.

## Conflict of Interest Statement

Nada Alachkar received one-time consultation reimbursement from Alexion Company in July 2012. Sami Alasfar declares no commercial or financial relationships that could be construed as a potential conflict of interest.
